# Easy xeno-free and feeder-free method for isolating and growing limbal stromal and epithelial stem cells of the human cornea

**DOI:** 10.1371/journal.pone.0188398

**Published:** 2017-11-17

**Authors:** Djida Ghoubay-Benallaoua, Céline de Sousa, Raphaël Martos, Gaël Latour, Marie-Claire Schanne-Klein, Elisabeth Dupin, Vincent Borderie

**Affiliations:** 1 Institut de la Vision, Sorbonne Universités, INSERM, CNRS UMR 7210, UPMC Univ Paris 06, Paris, France; 2 Centre Hospitalier National d’Ophtalmologie des Quinze-Vingts, Paris, France; 3 Etablissement Français du Sang–Ile-de-France, Paris, France; 4 Laboratoire Imagerie et Modélisation en Neurobiologie et Cancérologie, Univ. Paris-Sud, CNRS, Université Paris-Saclay, Orsay, France; 5 Laboratoire d'Optique et Biosciences, Ecole polytechnique, CNRS, INSERM U1182, Université Paris-Saclay, Palaiseau, France; Cedars-Sinai Medical Center, UNITED STATES

## Abstract

Epithelial and stromal stem cells are required to maintain corneal transparency. The aim of the study was to develop a new method to isolate and grow both corneal stromal (SSC) and epithelial limbal (LSC) stem cells from small human limbal biopsies under culture conditions in accordance with safety requirements mandatory for clinical use in humans. Superficial limbal explants were retrieved from human donor corneo-scleral rims. Human limbal cells were dissociated by digestion with collagenase A, either after epithelial scraping or with no scraping. Isolated cells were cultured with Essential 8 medium (E8), E8 supplemented with EGF (E8+) or Green’s medium with 3T3 feeder-layers. Cells were characterized by immunostaining, RT-qPCR, colony forming efficiency, sphere formation, population doubling, second harmonic generation microscopy and differentiation potentials. LSC were obtained from unscraped explants in E8, E8+ and Green’s media and were characterized by colony formation and expression of PAX6, ΔNP63α, Bmi1, ABCG2, SOX9, CK14, CK15 and vimentin, with a few cells positive for CK3. LSC underwent 28 population doublings still forming colonies. SSC were obtained from both scraped and unscraped explants in E8 and E8+ media and were characterized by sphere formation, expression of PAX6, SOX2, BMI1, NESTIN, ABCG2, KERATOCAN, VIMENTIN, SOX9, SOX10 and HNK1, production of collagen fibrils and differentiation into keratocytes, fibroblasts, myofibroblasts, neurons, adipocytes, chondrocytes and osteocytes. SSC underwent 48 population doublings still forming spheres, Thus, this new method allows both SSC and LSC to be isolated from small superficial limbal biopsies and to be primary cultured in feeder-free and xeno-free conditions, which will be useful for clinical purposes.

## Introduction

The cornea is a transparent window essential for vision, which forms the central part of the ocular surface [1]. The cornea is composed of three cell layers derived from two embryonic germ tissues: a stratified corneal epithelium of surface ectoderm origin, expressing the cytokeratins 3 and 12 (K3/K12), a stromal layer populated by keratocytes and composed of highly aligned collagen fibrils, and a monolayer of endothelial cells covering the posterior corneal surface [2, 3, 4]. The stromal and endothelial layers are derived from the cranial neural crest cells that migrate along the optic vesicles and home to the anterior eye region [5, 6, 7, 8, 9, 10].

Epithelial and stromal limbal stem cells, usually referred to as limbal stem cells (LSC) for epithelial cells and stromal stem cells (SSC) for stromal cells, are required to maintain corneal transparency [11]. Both stem cell types are located in the limbal niche [12].

Using full field optical coherence microscopy (FFOCM) coupled with a fluorescence channel, we have shown that LSC are localized in the limbal niche region at the bottom of the limbal crypts, which are located between the palisades of Vogt [13]. Through asymmetric division, one LSC generates a daughter LSC that contributes to the maintenance of the stem cell pool, and a transient amplifying cell (TAC) that migrates centripetally in the basal epithelial cell layer to the central cornea in order to replenish the corneal epithelium [14]. SSC are located in the corneal limbal region close to the epithelial LSC [12, 15]^.^ After injury of the corneal stroma, quiescent limbal stromal cells probably migrate from the limbal region to the site of injury. Stromal wound healing is a complex process involving cell death at the site of injury, migration of quiescent keratocytes followed by cell proliferation, differentiation and extracellular matrix synthesis and remodeling [16].

Both types of corneal stem cells are used in stem cell transplantation assays in animal models and in clinical trials aimed at restoring corneal epithelial function and stromal transparency [17, 18, 19]. Potential targets are various corneal disorders including limbal deficiency for LSC, keratoconus and other corneal ectasias, and corneal scars after infectious keratitis or trauma, for SSC. Furthermore, bioengineering technologies are currently developed, based on LSC and SSC, to prepare artificial cornea and limbal niche for transplantation [20, 21]. These artificial tissues would be of interest to replace conventional donor tissues. In fact, there is a lack of cornea donor tissue worldwide, since only one recipient out of 70 can be provided with a human donor tissue [22].

Several different culture methods have been used to grow human LSC [23]. Each of these methods begins with a small epithelial biopsy grown as explant or dissociated cells, with or without feeder cells or bovine serum [24, 25, 26]. Efforts have been made to investigate xenogeneic and feeder-free culture conditions [27, 28, 29, 30]. Small limbal biopsies (of 1-mm length) have been routinely retrieved from healthy donor eyes to isolate and grow LSC: either dissociated LSC are cultured with murine cell feeders, or LSC from limbal explants are grown on human amniotic membrane without feeders, before autologous transplantation in contralateral eyes presenting severe limbal deficiency [31, 32]. Hundreds of autologous cultured LSC transplantations have been performed in humans and small limbal biopsies taken in healthy donor eyes have been shown to be safe for the donor eye [33].

The present study aimed to devise a method to isolate and grow both LSC and SSC from such biopsies under xeno-free and feeder-free culture conditions in order to meet safety requirements that are mandatory for clinical use in humans.

## Materials and methods

### Isolation of human corneal progenitor cells

This study was carried out according to the tenets of the Declaration of Helsinki and followed international ethic requirements for human tissues. It was submitted to the Ethics Committee of the French Society of Ophthalmology (IRB 00008855 Société Française d’Ophtalmologie IRB#1) who waived approval for this type of study. Donor tissue procurement fulfilled all the legal requirements including absence of donor opposition to donation recorded in the French National Registry of Opposition and positive family testimony. None of the transplant donors were from a vulnerable population and all donors or next of kin provided written informed consent that was freely given.

Donor tissue consisted of either corneo-scleral rim obtained during surgery after 8-mm trephination of the graft or corneal grafts discarded during storage due to low endothelial cell counts. All tissues were processed by the EFS -Ile-de-France Cornea Bank (Paris, France). For corneal grafts, an 8-mm trephination was performed to isolate corneo-scleral rims. 6 human donor corneas, obtained between March 2016 and January 2017, were used to grow stem cells. The donor age ranged from 48 to 89 years with an average of 74 years.

Superficial limbal explants were prepared under a laminar flow. A stromal dissection between the anterior and mid-stroma was carried out using a 15° blade and sclera was carefully removed with scissors, resulting in superficial limbal rims that included limbal epithelium and superficial limbal stroma [34]. The obtained superficial limbal rims from six donor corneas, were either scraped with a scalpel to remove the limbal epithelium (three superficial limbal rims), when the aim was to obtain only stromal cells, or processed with no epithelial scraping (three superficial limbal rims), when both stromal and epithelial cells were expected. The rims were then cut into small pieces and incubated overnight at 37°C in 500μL of basal medium, that is, Dulbecco’s Modified Eagle’s Medium (DMEM, Life Technologies, Courtaboeuf, France) containing 1mg/mL collagenase A (Sigma Aldrich, Saint-Quentin Fallavier, France). Samples were then centrifuged and the supernatant was removed. One mL of DMEM was added and samples were centrifuged a second time followed by removal of the supernatant. Tissue debris was removed by filtration with a 100μm-pore diameter Macs SmartStrainers (Miltenyi Biotec, Paris, France). After centrifugation at 800g for 5 minutes (min), the cells were resuspended in DMEM and counted with a hemocytometer. Two types of isolated limbal cell (ILC) suspensions were obtained: cells prepared with epithelial scraping (ILC-scraping+) and cells prepared with no epithelial scraping (ILC-scraping-).

### Culture of isolated limbal cells (ILC) in different conditions

The isolated limbal cell suspensions (ILC-scraping+ and ILC-scraping-) were divided into three samples: one for culture with murine cell feeder-layers in Green’s medium, one for culture with no feeders in Essential 8 (E8) medium, and the last one for culture with no feeders in E8 medium supplemented with EGF (E8+).

#### Culture with 3T3 feeders in Green’s medium

Swiss albino murine 3T3 fibroblasts (ATCC, Molsheim, France) were treated with 4μg/ml mitomycin C (Sigma Aldrich, St Quentin Fallavier, France) for 2 hours (hr) and then trypsinized and plated at a density of 3x10^4^ cells per cm^2^ in 24-well culture plates. The medium used was cholera toxin-free Green medium [35]. It is made of a 3:1 mixture of calcium-free DMEM (Dutscher, Brumath, France) and Ham F12 medium (F12) (Life Technologies, Courtaboeuf, France), supplemented with 10% fetal bovine serum (FBS, Life Technologies), 1 mM/ml HEPES buffer (Life Technologies), 5 μg/ml human recombinant insulin (Actrapid^®^, Novo Nordisk, Paris, France), 0.4 μg/ml hydrocortisone (Pharmacia, Pfizer, Paris, France), 4 μM/ml L-glutamine (Life Technologies), 2 pM/ml tri-iodo thyronine (Sigma Aldrich), 200 nM/ml adenine (Sigma Aldrich), 100 IU/ml penicillin (Life Technologies), 100 μg/ml streptomycin (Life Technologies), 0.25 μg/ml amphotericin B (Life Technologies), 2 μg/ml isoproterenol (Hospira, Asnières, France) and 10 ng/ml human recombinant Epithelial Growth Factor (EGF) (Sigma Aldrich) [36]. Isolated limbal cells were seeded on feeder-layers of 3T3 fibroblasts (2x10^3^/ well) and cultured for 10 to 12 days at 37°C under 5% CO_2_. The culture medium was changed three times a week.

#### Culture with no feeders in E8 medium

Isolated limbal cells were seeded on 24-well culture plates at 10^4^ cells/well in E8 medium (Life Technologies). E8 Medium is a xeno-free and feeder-free medium especially formulated for the growth and expansion of human pluripotent stem cells (PSCs) [37, 38]. It is made of DMEM/F12, L-ascorbic acid, selenium, transferrin, NaHCO3, insulin, FGF2, and TGFß2. E8 medium was supplemented with 1.5% Methylcellulose gel matrix (Sigma Aldrich) to prevent re-aggregation of isolated cells [39]. Cells were grown for 21 days at 37°C under 5% CO_2_. The culture medium was changed three times a week.

To separate the two types of cells for analysis, the spheres, floating in the E8 medium, were isolated by aspirating the medium while the adherent spheres were scraped away with a cone. The well was washed twice with PBS. Adherent cells outside colonies were then removed with TrypLE (5 min) (Life technologies) followed by washing with PBS. Colonies of polygonal cells were then dissociated with TrypLE (10 min).

#### Culture with no feeders in E8 medium supplemented with EGF (E8+)

Isolated limbal cells were cultured in E8 medium supplemented with 10ng/ml of human recombinant EGF (Sigma Aldrich). Culture conditions were identical to those used for culture with no feeders in E8 medium, except for EGF supplementation of the medium.

### Clonal cultures of isolated limbal cells (ILC)

Three corneas from 3 different donors (donor age ranged from 77 to 89 years) were used for these experiments.

ILC-scraping+ were cloned by limiting dilution. A solution of 0.5 cells per 100μl of E8 medium was prepared. 100μl of this solution were plated in each well of 96-well plates. Following microscopic scoring after 24 hr of culture, only wells containing a single cell were used for further experiments. Cells were grown for two weeks, then enzymatically dissociated and serially subcultured at a density of 10^4^ cells per well until senescence.

ILC-scraping- were cloned by limiting dilution on a 3T3 feeder-layer in Green’s medium. After microscopic scoring at day 5 of culture, only wells containing one colony were used for further experiments. Cells were grown for 12 days, then enzymatically dissociated and serially subcultured at a density of 10^4^ cells per well until senescence.

The number of population doubling was determined at the end of each subculture and calculated with the following formula: log (N/N_0_)/log2, where N_0_ is the number of plated cells and N is the number of cells at the end of culture period.

### In vitro differentiation assays

#### Keratocyte and fibroblast differentiation

To promote differentiation of cultured cells into keratocytes, the cells dissociated from primary spheres cultured in E8 medium (using collagenase A 0.5mg/ml for 10 min at 37°C) or from adherent colonies (using TrypLE for 10 min at 37°C) cultured in E8 medium, were plated in DMEM/F12 supplemented with 200μM ascorbic acid (Sigma Aldrich). Fibroblastic differentiation was induced in DMEM/F12 supplemented with 10% FBS. After 14 days of culture, cells were fixed with 4% paraformaldehyde (PFA) for immunostaining.

#### Neuronal differentiation

For neuronal differentiation, individual primary spheres obtained after 14 days of culture or dissociated colonies, were transferred onto coverslips coated with 100μg/mL poly-D-lysine (Sigma Aldrich) and 50μg/mL laminin (Sigma Aldrich). Cells were cultured for 10 days in E8 medium supplemented with 1% FBS and 2% B27 (Life Technologies), then fixed for immunostaining.

#### Osteocyte, chondrocyte and adipocyte differentiation

To investigate the differentiation of dissociated spheres and colonies into mesenchymal cell lineages, we used three culture media developed to promote chondrogenic, osteogenic and adipogenic differentiation of mesenchymal stem cells (MSCs): StemPro® Chondrogenesis, Osteogenesis and Adipogenesis differentiation media, respectively (all from Life Technologies). MSCs from rat adult bone marrow were used as positive controls. Briefly, bone marrow was obtained from femur cavities of 8-week old Long Evans rats (Janvier Laboratories) after flushing in alpha-minimal essential medium, 10% FBS and 1% penicillin/streptomycin (Invitrogen, Carlsbad, CA, USA). Cells dissociated from the spheres and colonies as well as control MSCs were plated at a density of 10^5^ cells/cm^2^ in the appropriate differentiation medium. After 21 days, cultured cells were fixed with 4% PFA for 30 min, before staining with 1% Alcian Blue (Sigma Aldrich) for 30 min, with 2% Alizarin Red for 2 to 3 min (Sigma Aldrich) or with Oil Red O for 15 min (Sigma Aldrich).

#### Synthesis of organized extracellular matrix by stromal stem cells

After primary culture of ILC-scraping+ in feeder-free E8 medium, spheres were harvested and plated in an aligned nanofiber substrate in 24-well plates (Sigma Aldrich) in order to promote synthesis of an organized collagen extracellular matrix. After 3 weeks, cultures were fixed with 4% PFA, and examined by confocal microscopy after immunostaining or by multiphoton microscopy without staining.

#### Multiphoton microscopy

Multiphoton microscopy was performed using a custom-developed laser scanning upright microscope as previously described [40]. Two-photon excited fluorescence (2PEF) and second harmonic generation (SHG) images were recorded simultaneously in two different detection channels using appropriate spectral filters and they were combined using ImageJ software (NIH, Bethesda, MD, USA). These 2PEF/SHG images were obtained at increasing depths within the cells using a 25x, 1.05 NA objective lens with 0.4 μm (lateral) x 1.8 μm (axial) resolutions. Power at focus was 20 mW with 100 kHz pixel rate. The 2PEF images revealed endogenous fluorescence of the cells and SHG images specifically revealed collagen fibrils without any staining [41].

### Immunocytochemistry

Immunocytochemical analysis was performed in primary cultures of ILC-scraping+ and ILC-scraping- and after each in vitro differentiation assay. Briefly, cells were fixed with 4% PFA for 10 min, washed with PBS/Tween 0.25% (PBST), and incubated for 1 hour in PBS containing 0.3% Triton X100 and 0.2% gelatin (PBSGT) to block nonspecific binding. Cells were then incubated overnight at 4°C with primary antibodies diluted in PBST. After washing in PBS, cells were incubated for 1 hr at RT with the appropriate secondary antibodies diluted in PBST: Alexa Fluor 488 IgG (1:200) and Alexa Fluor 594 IgG (1:400) antibodies (Life Technologies). Nuclei were stained with Dapi (1:1000; Molecular Probes). Cells were washed 3 times, 5 min each with PBST followed by mounting with fluoromount-G (Clinisciences, Nanterre, France).

Primary antibodies used were: Human Natural Killer 1 (HNK1, pure hybridoma supernatant), SOX9 (1/400, Millipore, Saint-Quentin en Yvelines, France), SOX10 (1/300, Abcam, Paris, France), BMI1 (1/200, Abcam), PAX6 (1/100, Millipore), NESTIN (1/300, R&D systems, Lille, France), P63α (1/50, Cell signaling Technology, Leiden, The Netherlands), SOX2 (1/200, R&D systems), CK14 (1/100, Santa Cruz Biotechnology), CK15 (1/100, Santa Cruz Biotechnology), Cytokeratin 3 (CK3, 1/100, Santa Cruz Biotechnology, Heidelberg, Germany), LUMICAN (1/200, Sigma Aldrich), KERATOCAN (1/200, Santa Cruz), αSMA (1/800, Sigma Aldrich), VIMENTIN (1/200, Dakocytomation, Glostrup, Denmark), ßIII TUBULIN (1/200, R&D Systems), glial fibrillary acidic protein (GFAP, 1/500, Millipore), Tyrosine Hydroxylase (TH, 1/500, Millipore), COLLAGEN I (1/200, Sigma Aldrich), COLLAGEN IV (1/300, Millipore), COLLAGEN V (1/100, Abcam) and COLLAGEN 6A1 (1/300, Sigma).

Stained samples were observed with Olympus FV1000 laser-scanning confocal microscope (Olympus, Rungis, France). Twelve bit images were processed with ImageJ or FIJI, and Z-sections were projected on a single plane using maximum intensity under Z-project function. Images were finally converted in 24 bits RGB color mode and figures were then assembled.

### Quantitative polymerase chain reaction (qPCR)

Total RNA was isolated from primary cultured cells in E8 medium, using the ReliaPrep RNA cell Miniprep System (Promega, Charbonnières-les-Bains, France) according to the manufacturer’s instructions. The amount of total RNA isolated was quantified by optical density at 260 nm. cDNA was reverse transcribed from 1 to 2 mg of total RNA with Quantitect Reverse Transcription Kit (Qiagen, Hilden, Germany). Quantitative polymerase chain reaction (qPCR) of *CK3 (*Life Technologies, Cat# Hs00365074-m1), ΔN*P63α* (Life Technologies, Cat# Hs00978337-m1), *KERATOCAN* (Life Technologies, Cat# Hs00559942-m1), *PAX6* (Life Technologies, Cat# Hs01088114-m1) and *SOX2* (Life Technologies, Cat# Hs 01053049-s1) genes was carried out in a 20μL solution containing cDNA and TaqMan Gene Expression Assay Mix (Applied Biosystems, Courtaboeuf, France). The results of RT-qPCR were analyzed by the comparative threshold cycle method and normalized by using *18S RNA* (Life Technologies, Cat# Hs 9999991-s1) as an internal control. The amount of relative target gene mRNA was expressed with the following formula: CTgene—CT internal control = ΔCT and 1/ΔCT were calculated.

## Results

### Isolated limbal cell culture and separation of LSC and SSC

Human limbal cells from 6 donors were dissociated from superficial limbal explants, either after epithelial scraping or with no scraping. The isolated limbal cells (ILC-scraping+ and ILC-scraping-) were plated in three different culture conditions to compare their capacity for formation of spheres and adherent colonies: uncoated wells with Green’s medium and 3T3 feeders, E8 medium, and E8 medium supplemented with EGF.

**Fig 1:**
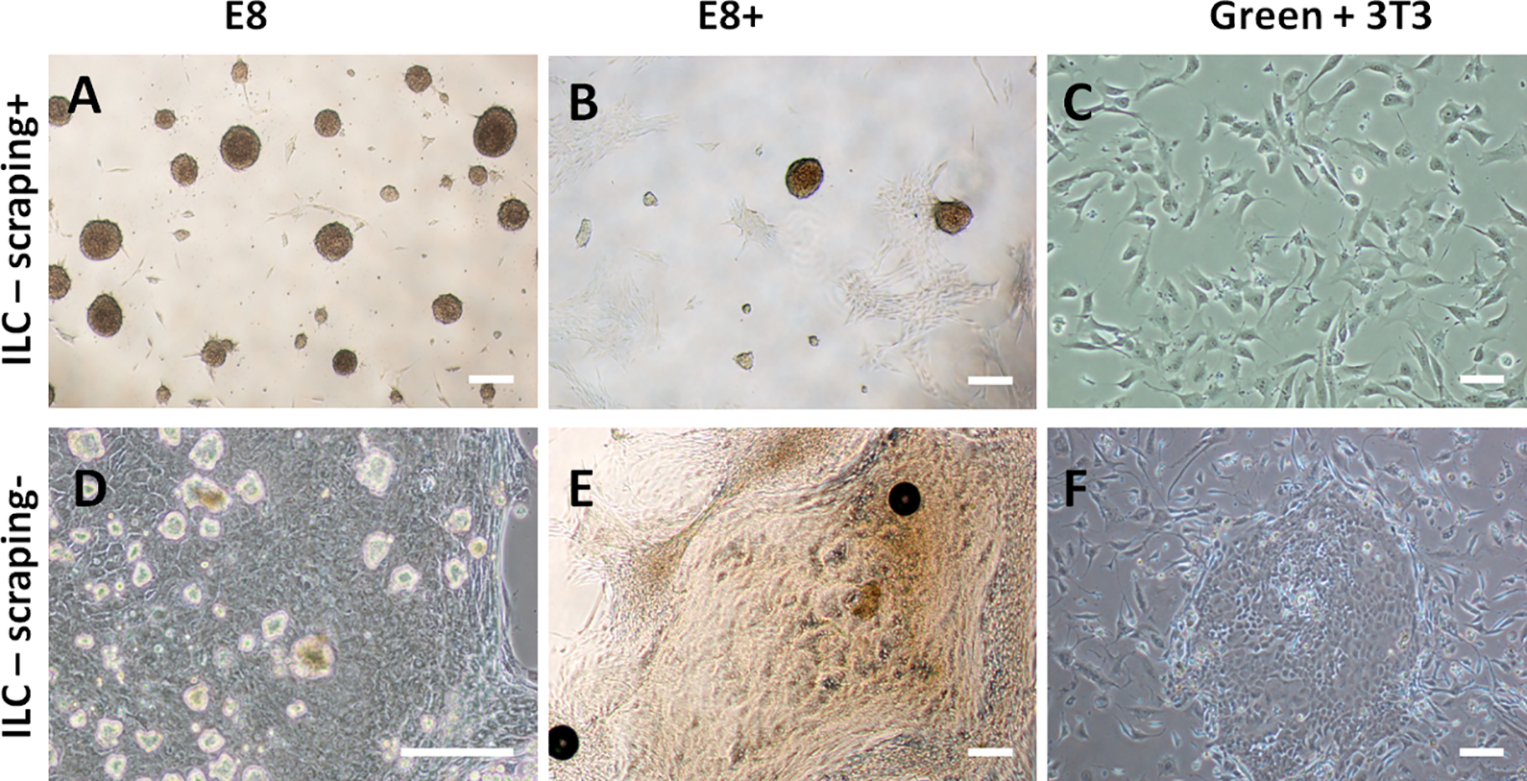
Colonies and spheres were obtained after primary culture of isolated limbal cells (ILC). ILC-scraping+ (A, B, C) and ILC-scraping- (D, E, F) were cultured in E8 (A, D), E8+ (B, E) and Green+ 3T3 (C, F) media. Spheres obtained after culture of ILC-scraping+ in E8 medium (A) are more numerous than in E8+ (B). More adherent cells are observed in E8+ than in E8 medium. No colonies are observed with ILC-scraping+ cultured in Green+3T3 medium and only 3T3 feeders are present (C). Culture of ILC-scraping- results in the presence of both spheres and colonies in E8 (D) and E8+ media (E). Colonies obtained in E8 medium are isolated and spheres float in the medium (D). Colonies obtained in E8+ medium are surrounded with cells (E). Colonies of polygonal cells with no spheres are observed with ILC-scraping- cultured in Green+3T3 medium (F). Bars, 200 μm.

In feeder-free E8 medium (E8) and E8 medium supplemented with EGF (E8+), ILC-scraping+ (Fig 1A and 1B) formed spheres that eventually floated free in the medium, and isolated adherent cells, but no colonies. Compared with E8, the E8+ condition resulted in a lower number of spheres (235±285/cm^2^ versus 657±256/cm^2^) and a higher number of adherent cells. ILC-scraping- (Fig 1D and 1E) formed spheres that eventually floated free in the medium, along with isolated adherent cells and few, albeit large individual colonies of polygonal cells, whereas the number of colonies appeared to be similar in E8 and E8+. In Green’s medium with 3T3 feeder layer, ILC-scraping+ did not grow, hence only 3T3 fibroblasts could be observed (Fig 1C), whereas ILC-scraping- formed small and numerous colonies of polygonal cells surrounded by 3T3 fibroblasts with no spheres (Fig 1F).

As the highest number of spheres was obtained with ILC-scraping+ grown in E8, this culture condition was used to produce stromal stem cells (SSC) in further experiments.

### Clonal growth of isolated SSC and LSC

**Fig 2:**
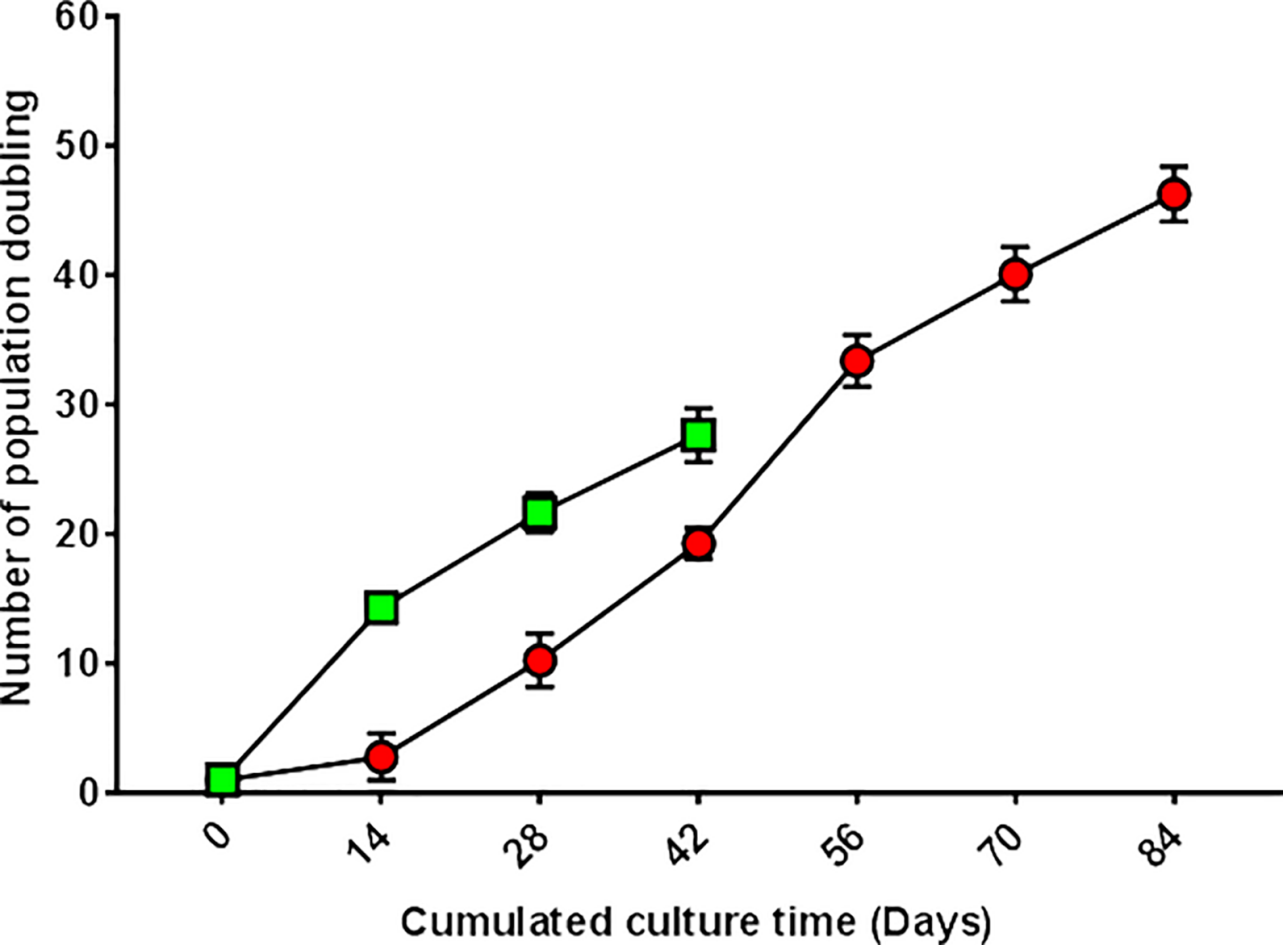
Growth potential of SSC and epithelial LSC. Isolated limbal cells (from 3 donors corneas) were cloned by limiting dilution. ILC-scraping**+** were plated in E8 medium and ILC-scraping**-** in Green+3T3 medium. Only wells containing 1 cell were used for culture. The potential of SSC (derived from ILC-scraping**+)** to grow until senescence was 48 population doublings (red), while the potential of LSC (from ILC-scraping-**)** was 28 population doublings (green) in these culture conditions.

To examine sphere formation and propagation potential of SSC, ILC-scraping+ were grown in E8 medium in limiting dilution assay. After 2 weeks, cultured cells formed spheres with a 1:1 ratio (i.e., 1 seeded cell formed 1 sphere). The average percentage of cells forming spheres was 4%. To evaluate the self-renewal capacity of SSC, primary spheres were dissociated and passaged under the same culture conditions. Secondary spheres were generated from dissociated primary spheres. In serial subculture, cloned cells could undergo 48 cumulative population doublings while maintaining their ability to form spheres (Fig 2). After 48 population doublings, cultured cells still divided but no longer formed spheres.

Epithelial LSC growth properties were investigated by limiting dilution assay using ILC-scraping- grown in Green’s medium with 3T3 feeder layer. After 12 days, cultured cells eventually formed colonies of polygonal cells with a 1:1 ratio, showing that each colony derived from a single cell. The average percentage of cells forming spheres was 1.3%. At confluency, each clone was dissociated and serially subcultured in the same culture conditions. Colonies were obtained at each passage until senescence characterized by cell growth arrest, which occurred after 28 population doublings (Fig 2).

### Stem cell and lineage marker expression in LSC and SSC cultures

**Fig 3:**
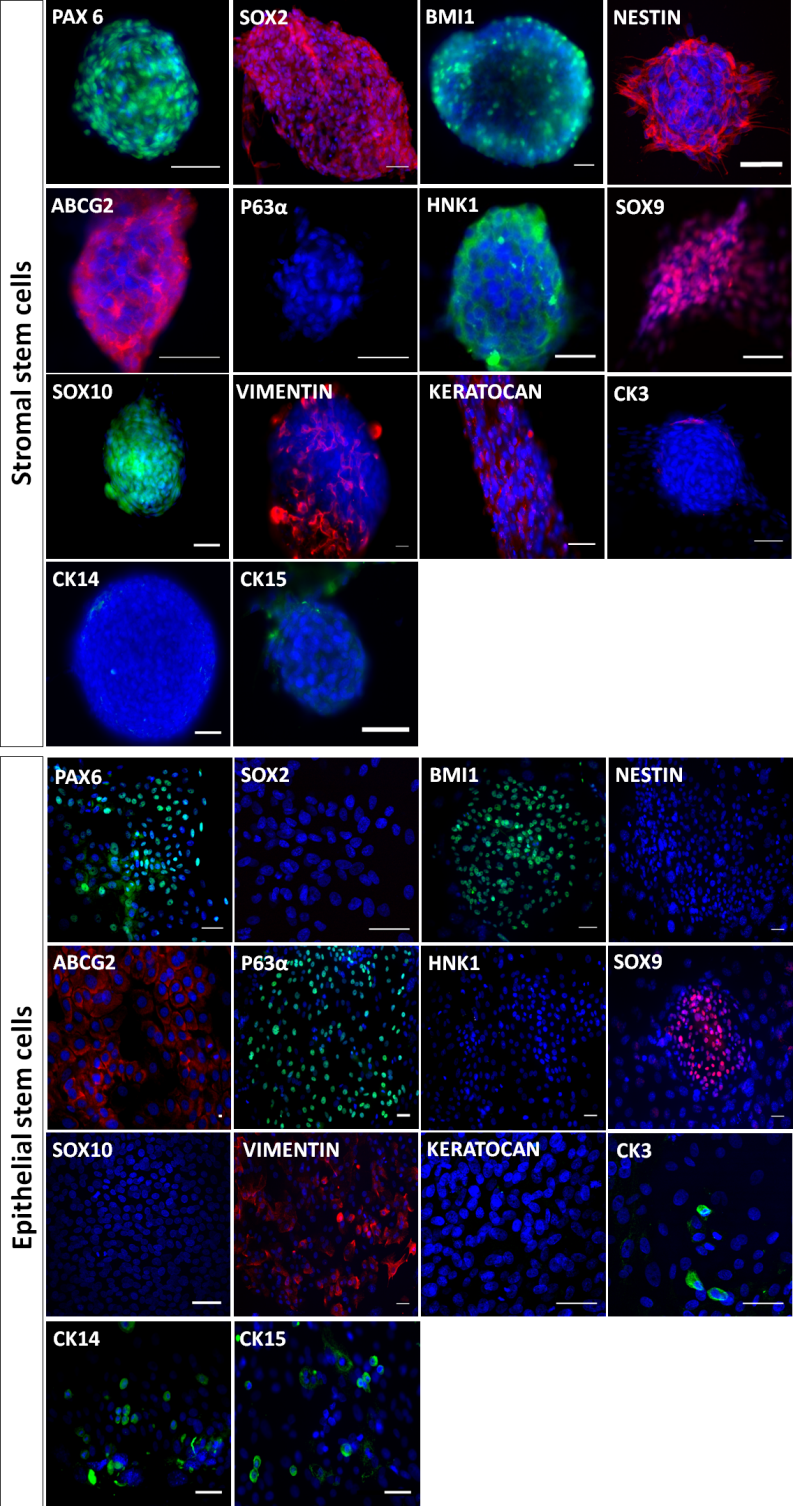
Marker analysis of SSC and LSC cultures by immunofluorescence. Stromal stem cells (spheres) were positive for all stem cell markers (PAX6, SOX2, BMI1, NESTIN and ABCG2) except P63α, CK14, CK15 and for keratocyte (VIMENTIN, KERATOCAN) and neural crest cell markers (HNK1, SOX9, SOX10) but negative for the corneal epithelial cell marker CK3. Epithelial limbal stem cells (colonies) were positive for the stem cell markers PAX6, BMI1, ABCG2, P63α, CK14 and CK15 but not for SOX2 and nestin. They featured low expression of the corneal epithelial cell marker CK3 and were Positive for SOX9 and vimentin but negative for HNK1, SOX10 and KERATOCAN. Bars, 50μm.

**Fig 4:**
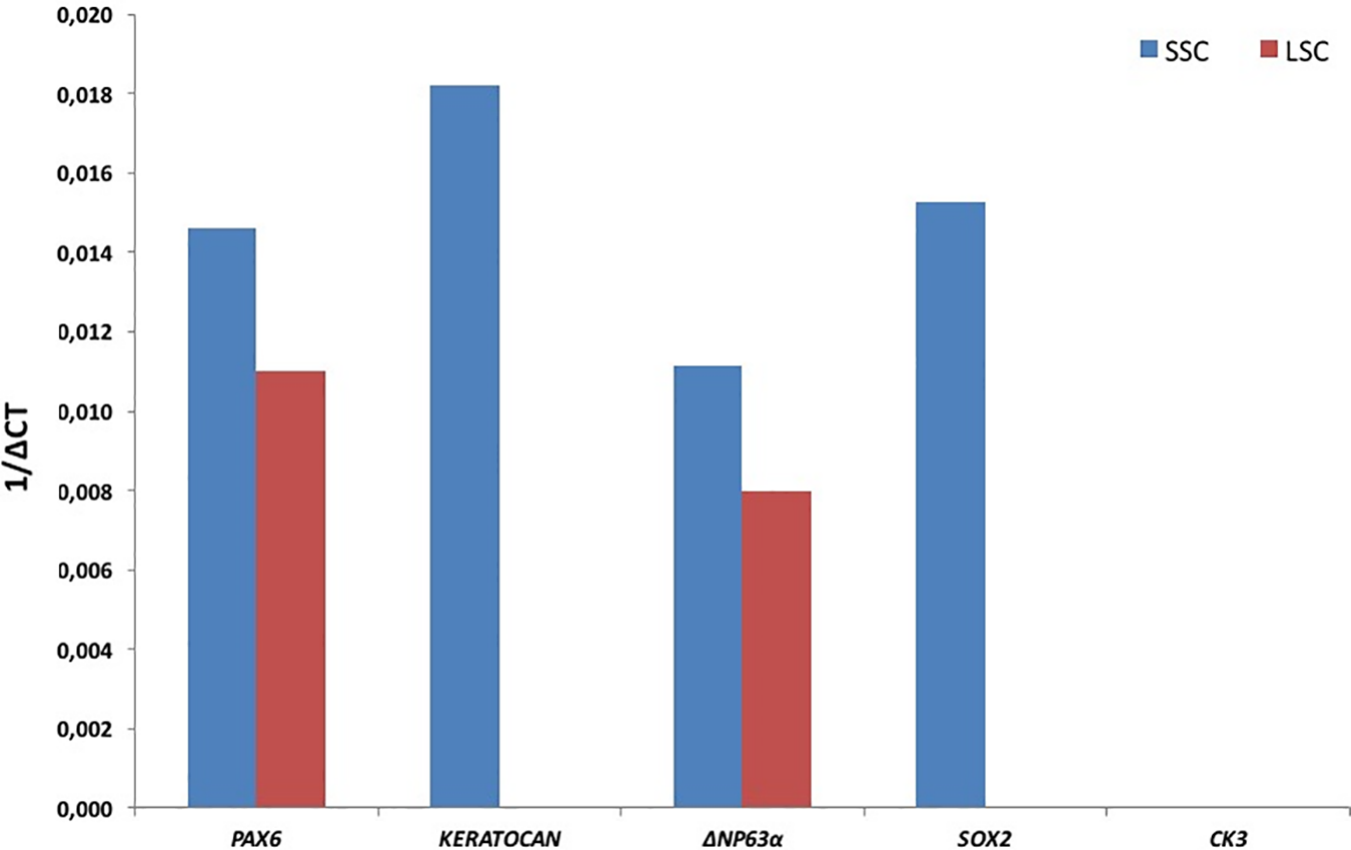
Marker gene expression in SSC and LSC by qRT-PCR analysis. SSC expressed *Pax6*, *keratocan*, *ΔNp63α*, and *SOX2* while expression of *CK3* was negative. LSC expressed transcripts of *PAX6* and *ΔNP63Α* but no transcripts for *KERATOCAN*, *CK3* and *SOX2* were detected in LSC.

To phenotypically characterize cells developing in spheres and colonies derived from limbal explants, we first examined the expression of various markers by immunofluorescence: markers for stem cells (i.e., PAX6, SOX2, BMI1, NESTIN, ABCG2, and P63α), neural crest cells (HNK1, SOX9 and SOX10), keratocytes (VIMENTIN and KERATOCAN) and corneal epithelial cells (CK3). As shown in Fig 3, spheres were positive for PAX6, SOX2, BMI1, NESTIN, ABCG2, HNK1, SOX9, SOX10, VIMENTIN and KERATOCAN, and negative for P63α, CK14, CK15 and CK3. The colonies of polygonal cells showed immunoreactivity to PAX6, BMI1, ABCG2, P63α, CK14, CK15, SOX9 and VIMENTIN, with a few cells positive for CK3; in contrast, they were negative for SOX2, NESTIN, HNK1, SOX10 and KERATOCAN. S1 Fig. shows staining of adherent cells obtained in E8+ medium. Adherent cells express PAX6, BMI1, NESTIN, ABCG2, VIMENTIN and KERATOCAN and they were negative for SOX2, P63α, HNK1, SOX9, SOX10 and CK3. In addition, expression of some of these marker genes was assessed by qPCR analysis of the spheres and colonies. Transcripts of *PAX6*, *KERATOCAN*, *ΔNP63Α*, and *SOX2* were detected in SSC-derived spheres while the transcripts for *CK3* were not detected (Fig 4). Primary colonies of epithelial polygonal cells expressed transcripts for *PAX6* and *ΔNP63Α* whereas transcript for *CK3*, *SOX2* and keratocan were not detected.

### Synthesis of collagen extracellular matrix by stromal stem cells

**Fig 5:**
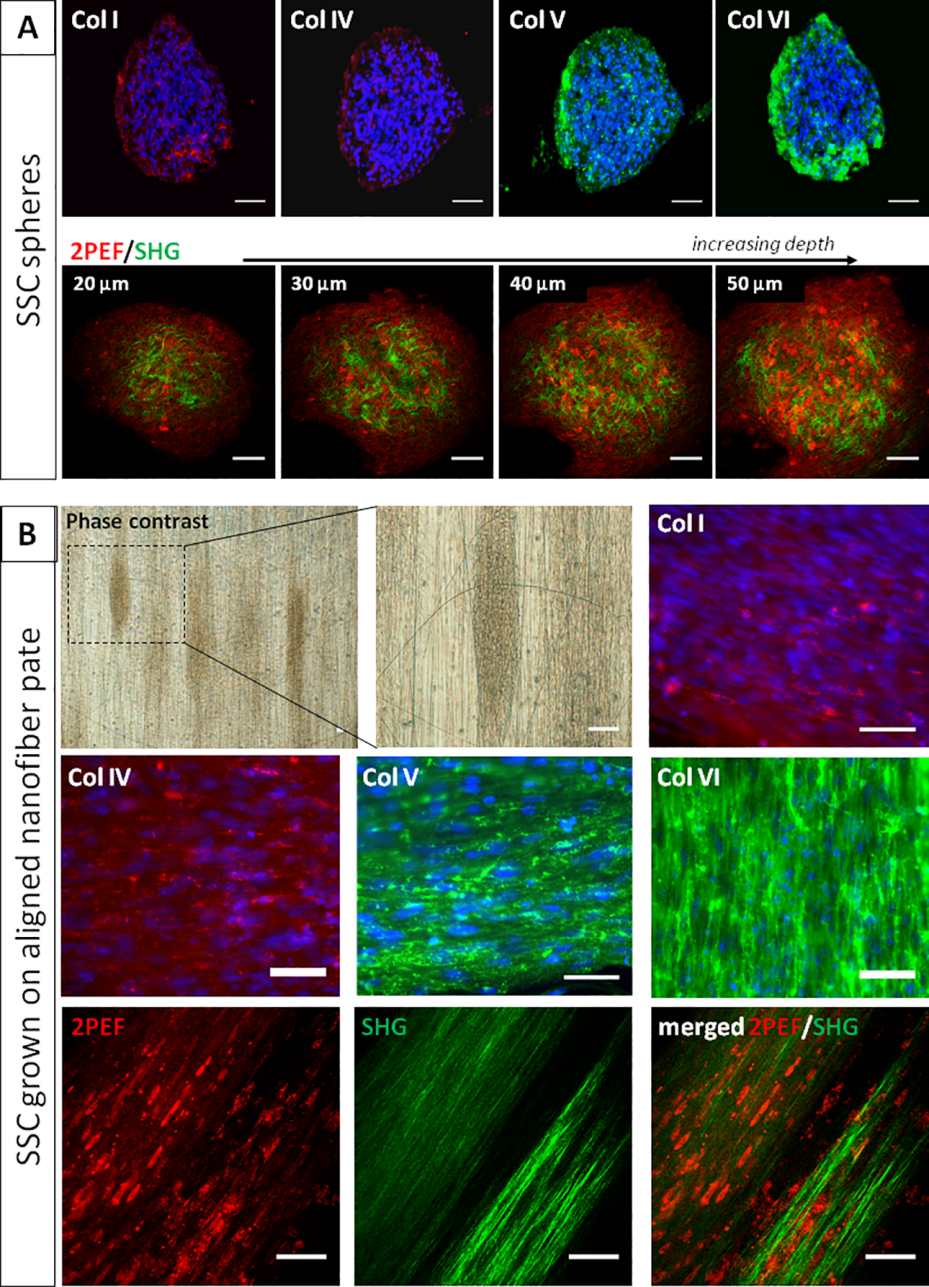
Collagen synthesis by cultured SSC. (A) Spheres derived from SSC produce collagen type I, IV, V and VI (as shown by immunohistochemistry), with no apparent organization. As shown by multiphoton microscopy, these disorganized collagen fibrils (SHG signals in green) are present around cells (endogenous fluorescence, 2PEF in red) along the full depth of spheres of SSC. (B) When spheres of SSC were plated on nanofiber plates to promote synthesis of an organized collagen extracellular matrix, spheres tended to elongate along nanofibers (as shown by phase contrast microscopy). The same feature was evident after immunostaining of collagen type I, IV, V and VI. Aligned collagen fibrils (green) were also evidenced by SHG microscopy. Bars, 50 μm.

After primary culture, spheres of SSC produced collagen type I, IV, V and VI with no preferential organization as revealed by immunofluorescence staining of collagens, which was confirmed by SHG microscopy (Fig 5A). In order to promote synthesis of organized collagen fibrils, the spheres of SSC were plated on aligned nanofiber multiwell plates. Phase contrast light microscopy showed that spheres then exhibited an elongated shape parallel to nanofibers. The collagen fibrils were also orientated along the nanofibers as revealed by immunofluorescence staining and polarization-resolved SHG microscopy (Fig 5B).

### Differentiation potentials of sphere forming SSC and colony forming LSC

After primary culture of ILC-scraping- and ILC-scraping+, spheres and colonies were dissociated and cultured in different differentiation promoting media as described in Materials and Methods.

### Keratocyte and fibroblast differentiation

**Fig 6:**
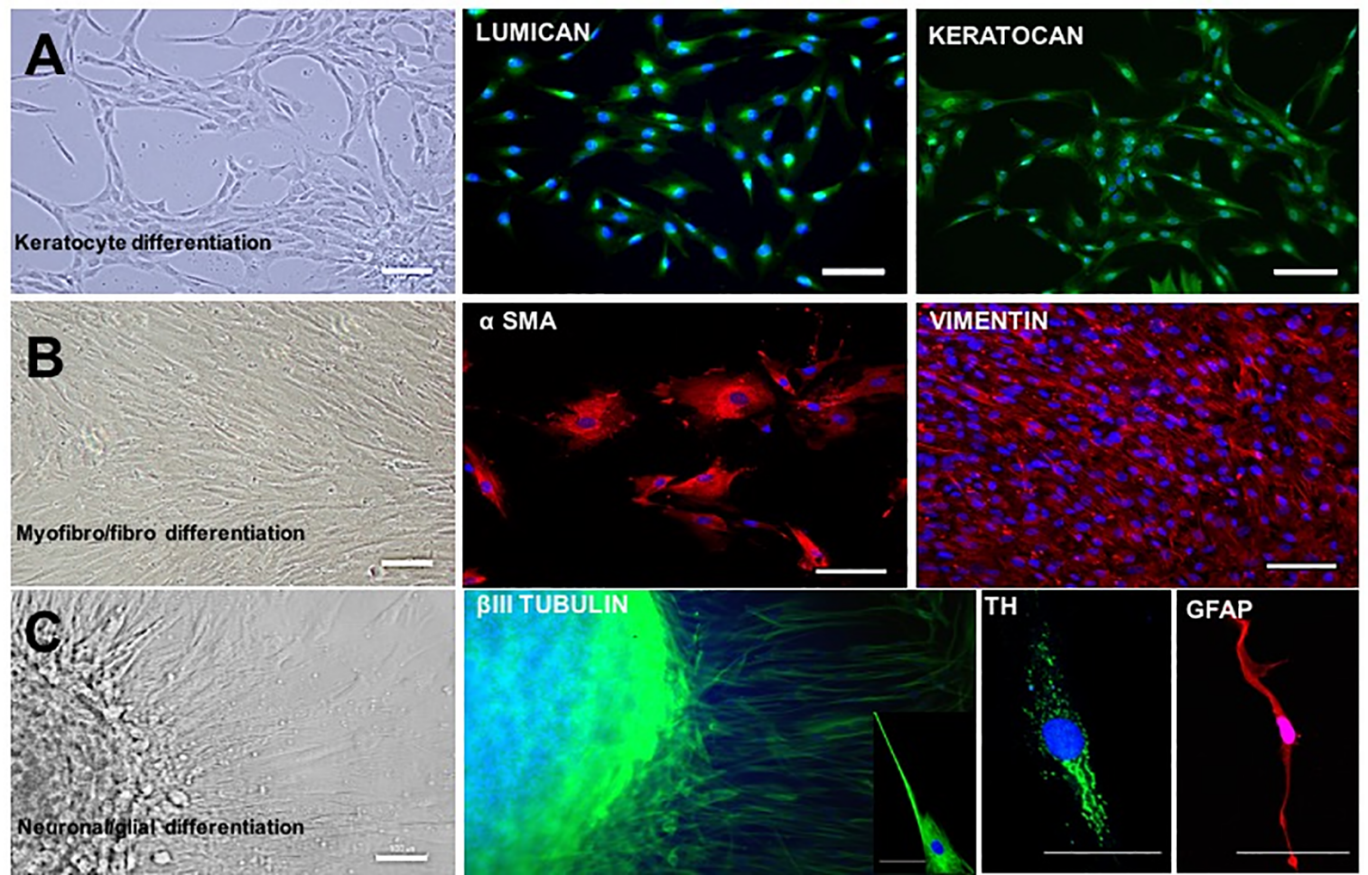
Keratocyte, myofibroblasts/fibroblasts, neuronal and glial differentiation of SSC. When exposed to differentiating medium conditions, SSC are able to differentiate into keratocytes, myofibroblasts, fibroblasts and neurons. Culture in keratocyte specific medium results in cells with dendritic morphology expressing LUMICAN and KERATOCAN (A). In DMEM medium supplemented with 10% serum, cells present a myofibroblastic (large cells) and fibroblastic (elongated cells) morphology and they express α-SMA and VIMENTIN respectively (B). Neuronal cells were present with elongated nerve fibers (C) and expression of βIII TUBULIN and Tyrosine Hydroxylase (TH); glial cells were detected by expression of GFAP (C). Bars, 50μm.

After 14 days of culture in keratocyte differentiation medium, cells with dendritic morphology were observed (Fig 6A). They were positive for LUMICAN and KERATOCAN immunostaining (Fig 6A). In basal medium containing 10% FBS, cells displayed features of fibroblasts and myofibroblasts, including expression of VIMENTIN and α-SMA proteins (Fig 6B).

In contrast, cells from dissociated colonies of LSC seeded in the keratocyte and the fibroblast differentiation medium did not adhere and no cell growth was observed (S2A and S2B Fig).

### Neuronal and glial differentiation

Individual spheres obtained after primary culture of limbal cells in E8 medium, were plated in poly-D-lysine and laminin coated wells. Ten days after plating, cells exhibited a neuronal morphology and staining for the neuronal markers βIII TUBULIN and TH (Fig 6C). Cultures also contained glial cells positive for GFAP (Fig 6C).

When colonies of LSC were dissociated, and seeded in the same medium, cells did not adhere and no cell growth was observed (S2C Fig).

### Trilineage mesenchymal differentiation

**Fig 7:**
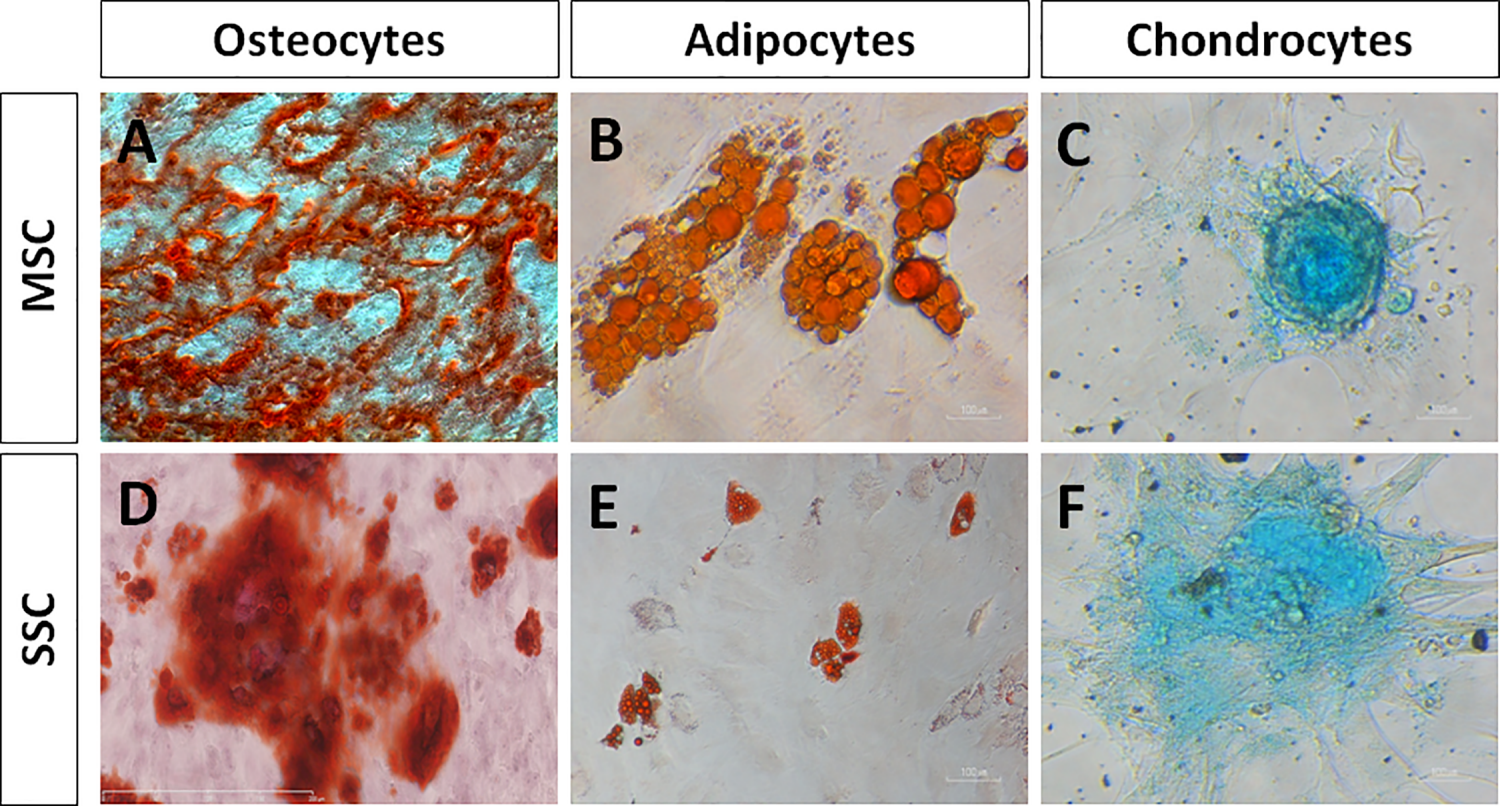
Mesenchymal cell differentiation assays of SSC. After primary culture, MSC (used as positive controls) and SSC were dissociated and plated into osteocyte, adipocyte and chondrocyte differentiating media. MSC (A-C) and SSC (D-F) show the ability to differentiate into osteocytes identified by calcium deposits (A, D; Alizarin Red staining), adipocytes (B, F; lipids stained by Oil RedO) and chondrocytes (C, F; matrix proteoglycans revealed by Alcian Blue staining). Bars, 100μm.

SSC and LSC switched to adipogenic, chondrogenic or osteogenic differentiation media were assayed for mesenchymal phenotypes after 3 weeks in culture (Fig 7). MSC cultured in the same conditions were used as positive controls. The staining of SSC cultures showed that SSC had the ability to differentiate into osteocytes (Alizarin Red staining) (Fig 7D), adipocytes (Oil RedO staining) (Fig 7E), and chondrocytes (Alcian Blue staining) (Fig 7F). By contrast, LSC were not able to differentiate into osteocytes, adipocytes and chondrocytes (S2D, S2E and S2F Fig).

## Discussion

From small superficial limbal biopsies taken from human donor corneas, we have developed culture methods whereby both LSC and SCC could be isolated with the same medium in primary culture. In E8 medium, scraping of limbal explants resulted in growth of isolated SSC without LSC, whereas cultures derived from non-scraped limbal explants resulted in growth of both LSC and SSC in the same well. LSC and SSC could be separated after removal of SSC by aspirating floating spheres and dissociating adherent ones. Following primary culture, both populations could be further grown as pure cell lineages, by growing LSC in Green’s medium with 3T3 feeders, and SSC in E8 medium.

E8 medium has been used previously to grow induced pluripotent stem cells (iPS) and permitted the growth of iPS in feeder-free and xeno-free culture condition [35, 42]. E8 medium is commercially available; this is of interest for cell therapy units aimed to standardize method preparation of cells for transplantation in patients. Compared with the medium described by others for the culture of SSC, E8 medium is a xeno-free medium, which does not contain FBS [43, 44], B27 supplement [45, 46] and cholera toxin [42]. To our knowledge, E8 medium has never been used to grow limbal epithelial and stromal stem cells. Our results show that E8 medium allows adult SSC to grow with maintenance of stemness through 48 population doublings together with various differentiation potentials. Sixty-five to 67 cumulative populations doubling of SSC isolated from fresh bovine corneas were found by Funderburgh et al. [47]. As we used elderly donor tissues as a source of isolated limbal cells, the growth potential of our cells was probably limited, although still valuable for therapeutic developments. Interestingly, elderly human donor tissue is routinely used for transplantation, provided the corneal endothelial cell density is higher than 2000 cells/mm2. Results of such transplantations of elderly donor corneas are successful [48].

Isolation and expansion of both SSC and LSC in the same culture condition (i.e., ILC-scraping- grown in E8 medium) may lead to cross contamination of expanded cells. This issue is supported by previous studies [49]. This group found that compared with dispase-isolated sheets, LSC outgrowth generated by collagenase-isolated clusters featured larger diameter and its single cells yielded more holoclones on 3T3 fibroblast feeder layers. The collagenase-isolated clusters were shown to not only consist of LSC but also their closely associated SSC [49]. In the present study, after primary culture of collagenase-isolated cells, subculture of spheres resulted in growth of both adherent cells and spheres featuring SSC phenotype and no colonies of epithelial cells. No CK3 and p63alpha positive cells were observed in these secondary cultures. Similarly, cultures of epithelial cells retrieved from primary culture colonies grown with 3T3 feeders in Green’s medium resulted only in colony formation with no spheres. Cells grown in this latter secondary culture condition featured positive staining for LSC markers (CK3/p63 alpha) and negative staining for SSC markers (HNK1, SOX10 and KERATOCAN)

In the present work, the two stem cell populations grown from limbal explants exhibited clear-cut differences regarding phenotype, growth potential and differentiation ability. SSC featured expression of SOX2, NESTIN, HNK1, SOX10 and KERATOCAN, absence of P63α CK14, CK15 and CK3 expression, high growth rate, and the capacity to differentiate into various cell lineages, including keratocytes, myo/fibroblasts, neurons, osteocytes, chondrocytes and adipocytes, with no epithelial differentiation potential. In contrast, LSC featured expression of P63α, CK14, CK15 and CK3 markers, absence of expression of SOX2, NESTIN, HNK1, SOX10 and KERATOCAN, lower growth potential and a unique epithelial differentiation potential. Both stem cell populations expressed PAX6 (these results were also described by others [45, 50, 51], ABCG2, BMI1, SOX9 [43], and VIMENTIN [47] [47]. Our results show that isolated human SSC expressed neural crest markers, displayed stem cell characteristics and were multipotent. They were also able to form spheres. As opposed to SSC, LSC expressed ectoderm together with stem cell markers and were unipotent in our culture conditions. Although they originated from the same donors, the two stem cell populations showed different growth potentials. This could be explained by the very rapid turnover of the corneal epithelium, which probably requires frequent LSC divisions. In a rabbit experimental model, the renewal time of the corneal epithelium has been shown to be 2 to 3 weeks under physiologic conditions [52]. Conversely, corneal keratocytes are thought to divide less frequently and SSC divisions are probably less frequent. However, we still lack clinical and experimental data concerning precise *in vivo* assessment of keratocyte turnover. Interestingly, macular dystrophy, which is a genetic corneal disorder with stromal deposits produced by keratocytes, presents rare and slow recurrence after corneal transplantation whereas keratoepithelin-linked dystrophies, which are genetic corneal disorders with corneal deposits originating mainly from the corneal epithelium, exhibit quick and constant recurrence [53, 54].

Due to a lack of donor cornea worldwide, development of alternative therapeutics has become an important goal. The study of extracellular matrix deposition by SSC *in vitro* serves to initiate the production of stroma-like tissue. Here, collagen deposition in cultured SSC spheres was revealed by whole mount immunohistochemical analysis and SHG examination. SHG showed that assembled collagen fibrils, not only collagen molecules, were synthesized by SSC. Immunohistochemical analysis revealed the presence of COLLAGEN type I, IV, V and VI, the main components of the human corneal stroma. Furthermore, fibril orientation could be obtained by growing cells on nanofiber plates. In previous studies, a highly aligned PEUU [poly(ε-caprolactone)-based poly (ester urethane) urea] nano-fibrous substrate served as a template to initiate and guide the organization of corneal stroma-like tissue from human corneal SSC. The addition of FGF-2 and TGF-β3 successfully stimulated the production of a stroma-like tissue comprised of multilayered lamellae with orthogonally oriented collagen fibrils and an abundance of cornea-specific proteins and proteoglycans [55, 56, 57].

## Conclusion

In conclusion, we show that both human adult LSC and SCC can be isolated from small superficial limbal biopsies and primary cultured in the same feeder-free and xeno-free culture conditions. These cultured stem cells feature high growth potential and the expected phenotypes and differentiation potentials in primary culture. Whereas SSC can be subcultured in the same feeder-free and xeno-free conditions LSC still need 3T3 feeders to be subcultured and further developments are needed to maintain their phenotypes after several passages in E8 medium. In case of autologous transplantation, the biopsy required to obtain limbal explants is known to be minimally invasive with no clinical consequences for the donor eye. It can be taken from a human donor cornea in case of allogeneic transplantation. We report a novel method for the isolation of both epithelial and stromal stem cell populations and their primary culture in feeder-free and xeno-free conditions, which will be of significant help for further cell therapy developments.

## Supporting information

S1 Fig Marker analysis of adherent cells (E8 medium) by immunofluorescence. Adherent cells were positive for PAX6, BMI1, NESTIN, ABCG2 except SOX2 and P63α, positive for keratocyte markers (VIMENTIN, KERATOCAN) and negative for neural crest markers (HNK1, SOX9, SOX10) and epithelial cell markers CK3. Bars, 50μm.
(TIFF)

S2 Fig Differentiation potential of LSC. Colonies of LSC obtained after primary culture in E8 medium were seeded in keratocyte, fibroblast and neuronal differentiation media, or the culture medium was switched to adipocyte, chondrocyte and osteocyte differentiation media. LSC did not adhere in keratocyte (A), fibroblast (B) and neural (C) differentiation medium and no growth was obtained. No differentiation to the mesenchymal lineages was observed for LSC (D, E and F). Bars, 100 μm.
(TIFF)
