# Supplementary figures and images for: Easy xeno-free and feeder-free method for isolating and growing limbal stromal and epithelial stem cells of the human cornea
Source: PLoS One. 2017 Nov 17;12(11):e0188398. doi: 10.1371/journal.pone.0188398 (PMC5693460; doi:10.1371/journal.pone.0188398)

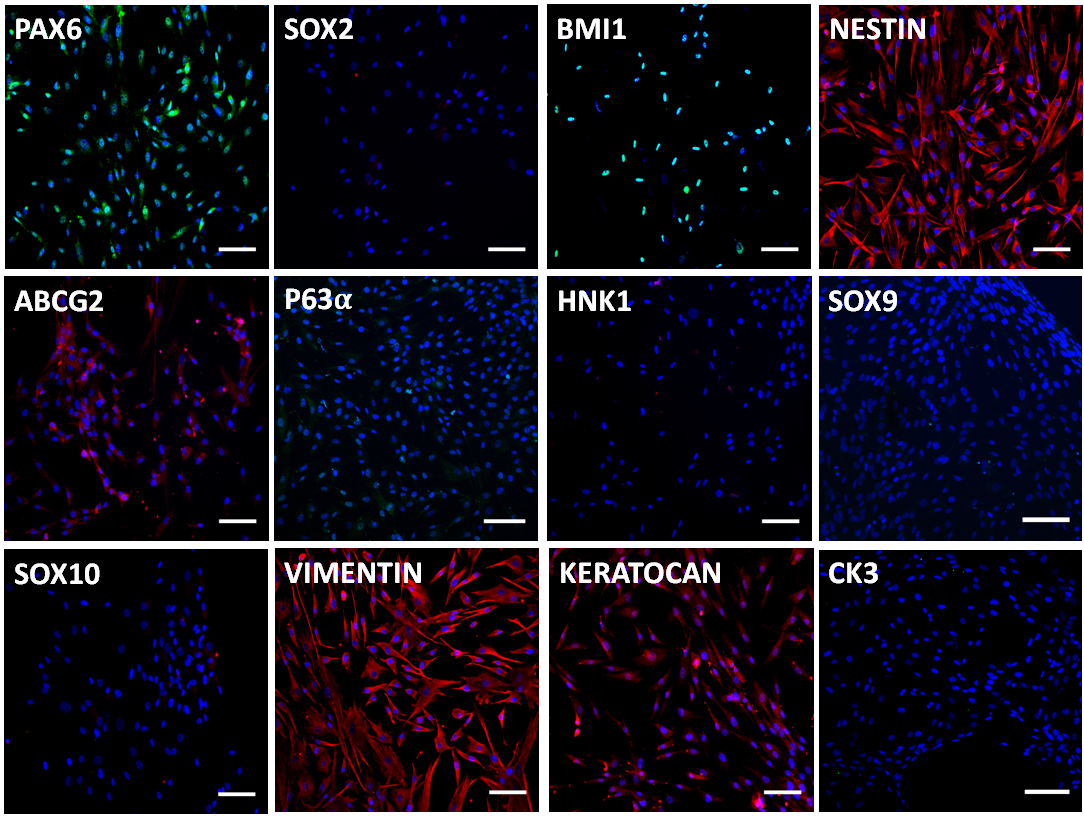

Supplement: S1 Fig — Adherent cells were positive for PAX6, BMI1, NESTIN, ABCG2 except SOX2 and P63α, positive for keratocyte markers (VIMENTIN, KERATOCAN) and negative for neural crest markers (HNK1, SOX9, SOX10) and epithelial cell markers CK3. Bars, 50μm. (TIFF) [file pone.0188398.s001.tiff]

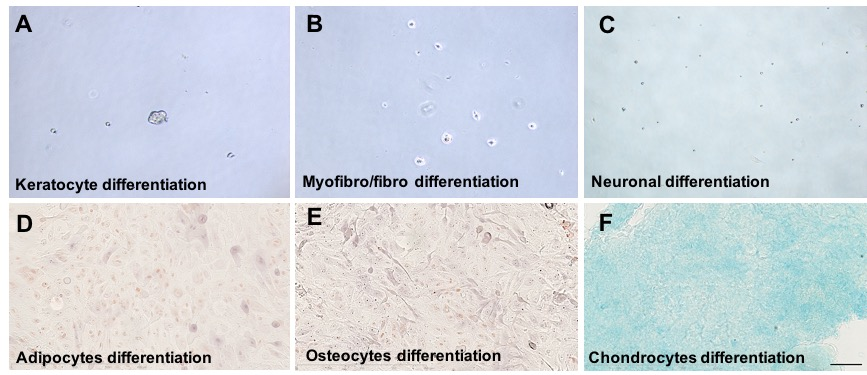

Supplement: S2 Fig — Colonies of LSC obtained after primary culture in E8 medium were seeded in keratocyte, fibroblast and neuronal differentiation media, or the culture medium was switched to adipocyte, chondrocyte and osteocyte differentiation media. LSC did not adhere in keratocyte (A), fibroblast (B) and neural (C) differentiation medium and no growth was obtained. No differentiation to the mesenchymal lineages was observed for LSC (D, E and F). Bars, 100 μm. (TIFF) [file pone.0188398.s002.tiff]
